# Integration of pH Control into Chi.Bio Reactors and Demonstration with Small-Scale Enzymatic Poly(ethylene terephthalate) Hydrolysis

**DOI:** 10.1021/acs.biochem.4c00149

**Published:** 2024-06-22

**Authors:** Mackenzie C. R. Denton, Natasha P. Murphy, Brenna Norton-Baker, Mauro Lua, Harrison Steel, Gregg T. Beckham

**Affiliations:** † Renewable Resources and Enabling Sciences Center, National Renewable Energy Laboratory, Golden, Colorado 80401, United States; ‡ BOTTLE Consortium, Golden, Colorado 80401, United States; § Catalytic Carbon Transformation and Scale-up Center, National Renewable Energy Laboratory, Golden, Colorado 80401, United States; ∥ Department of Engineering Science, University of Oxford, Oxford OX1 3PJ, U.K.

## Abstract

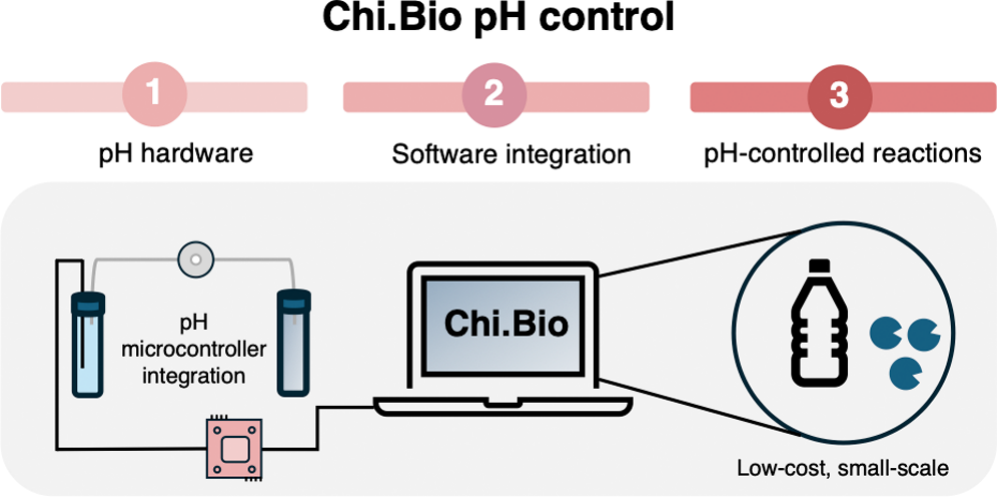

Small-scale bioreactors that are affordable and accessible would be of major benefit to the research community. In previous work, an open-source, automated bioreactor system was designed to operate up to the 30 mL scale with online optical monitoring, stirring, and temperature control, and this system, dubbed Chi.Bio, is now commercially available at a cost that is typically 1–2 orders of magnitude less than commercial bioreactors. In this work, we further expand the capabilities of the Chi.Bio system by enabling continuous pH monitoring and control through hardware and software modifications. For hardware modifications, we sourced low-cost, commercial pH circuits and made straightforward modifications to the Chi.Bio head plate to enable continuous pH monitoring. For software integration, we introduced closed-loop feedback control of the pH measured inside the Chi.Bio reactors and integrated a pH-control module into the existing Chi.Bio user interface. We demonstrated the utility of pH control through the small-scale depolymerization of the synthetic polyester, poly(ethylene terephthalate) (PET), using a benchmark cutinase enzyme, and compared this to 250 mL bioreactor hydrolysis reactions. The results in terms of PET conversion and rate, measured both by base addition and product release profiles, are statistically equivalent, with the Chi.Bio system allowing for a 20-fold reduction of purified enzyme required relative to the 250 mL bioreactor setup. Through inexpensive modifications, the ability to conduct pH control in Chi.Bio reactors widens the potential slate of biochemical reactions and biological cultivations for study in this system, and may also be adapted for use in other bioreactor platforms.

There is a major global effort underway to automate, down-scale, and democratize biological research. As a key component of these efforts, both commercial and open-source equipment are being developed for microbial cultivation and execution of biochemical reactions in a miniaturized context with online monitoring, with the intent to greatly accelerate the rate of data generation at lower cost, in reduced physical space, and with lower materials consumption.^1−6^ Moreover, to make biological research more accessible, there is a substantial drive for open-source hardware and software in biological research that often follows a do-it-yourself (DIY) model, enabled by the assembly of low-cost, off-the-shelf components.^7−9^ Of particular interest for biotechnological applications, commercial bioreactors with built-in control systems are usually quite costly, restricting their purchase primarily to companies and well-funded research laboratories. As a result, there have been many efforts to enable greater access to bioreactor hardware and software that, taken together, can result in the same or higher throughput than commercial bioreactors at 1–2 orders of magnitude cheaper, and using open-source software and DIY components.^10−17^

Of relevance to the current work, the Chi.Bio system was introduced by Steel et al. in 2020 as an open-source bioreactor system that enables continuous bioprocess monitoring, spectrometry, light outputs, among other features.^16^ Steel et al. demonstrated the use of the parallelized Chi.Bio system in assays for cell growth, the formation of biofilms, control over optogenetics systems, and the simultaneous readout of orthogonal fluorescent protein signals. Conveniently, the Chi.Bio system is available both for purchase as parts that can be assembled, or the entire unit is available for purchase, which at the time of writing is for $990 for a control computer, reactor, and pump board. For systems like Chi.Bio, which can conduct both biochemical reactions and whole-cell cultivations, the ability to both continuously monitor and control the pH could expand the utility of this system to experimental systems where substrates or products that modify the pH of an aqueous medium dynamically vary.

To that end, here we integrate pH control into the Chi.Bio system through the modification of both the hardware, using off-the-shelf components, and through modifications to the open-source Chi.Bio software. We validate the ability of the Chi.Bio system to monitor and control pH using enzymatic hydrolysis of poly(ethylene terephthalate) (PET) as a demonstration application, and compare this to enzyme performance in a commercial 1 L bioreactor with an initial working volume of 250 mL. pH-stats have been widely adopted to monitor the kinetics and conversion extents of the enzymatic degradation of polyesters and are an excellent means to benchmark activity.^18,19^ Overall, this work has a wide variety of potential applications across any process development involving pH-controlled biocatalytic reactions.

## Results

### Hardware Integration of pH Control Functionality to Chi.Bio Reactors

**Figure 1:**
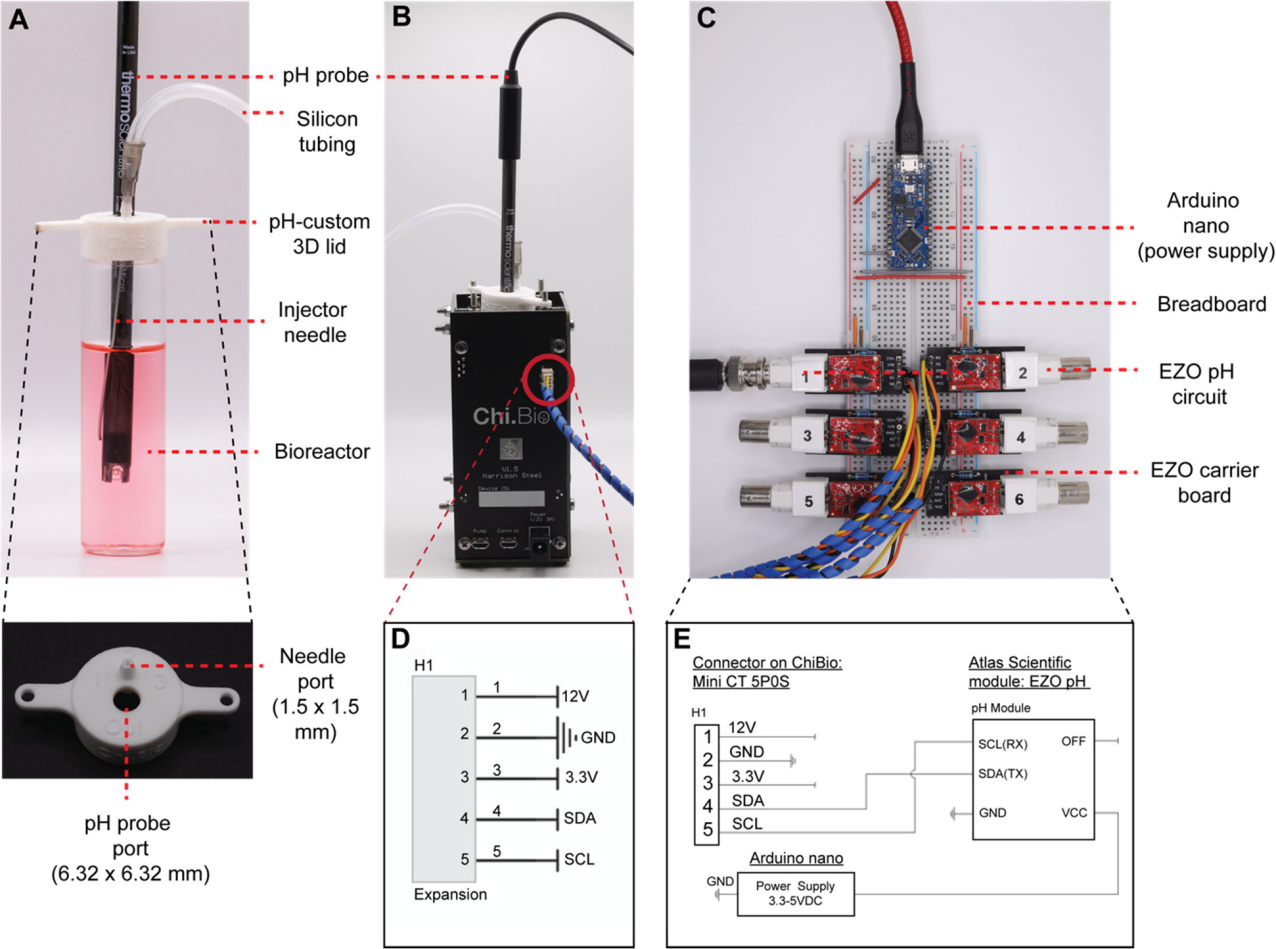
The hardware system for pH control integrated into Chi.Bio. The hardware modifications comprise an (A, B) off-the-shelf pH probe sourced from ThermoScientific (Orion Economy Series pH Combination Electrode #911600), inserted through a port in the custom 3D-printed head plate, the existing Chi.Bio peristaltic pump board (not shown), 2.5 mm × 1.0 mm silicon laboratory tubing (AlteSil High Strength Tubing, Altec, #01–93–1416), connected to an Air-Tite Premium Hypodermic Lab/Vet Use needle, and (C) an off-the-shelf Atlas Scientific pH circuit (EZO pH Circuit #EZO-pH) integrated to an Electrically Isolated EZO Carrier Board (#ISCCB-2). (D) Wiring diagram of expansion of the Chi.Bio Main Unit with the expansion header highlighted by red circle. (E) Wiring diagram of the Atlas Scientific EZO pH Module and ChiBio interface where all ground pins are connected to common ground. The 3.3 V, 12 V, and OFF pins are not connected.

To realize a platform that enables pH monitoring and continuous pH control by acid or base addition, we modified the existing Chi.Bio system hardware (Labmaker). A list of all individual hardware components of the pH control module can be found in the Supporting Information (SI, Table S1). The modifications comprise a customized head plate 3D-printed using a Stratasys Fortus 450MC printer with a 6.32 mm diameter port for insertion of a pH probe and a 1.5 mm diameter cut-out for an injector needle (CAD file, Supporting Information). A single silicon tubing line was connected to the injector needle, which inserts through the needle port of the customized head plate into the Chi.Bio reactor to provide a physical link for chemical addition back to the peristaltic pump board (Figure 1A). An off-the-shelf pH probe (ThermoScientific) was inserted centrally into the reactor via the headplate to enable real-time pH monitoring (Figure 1B). The pH probe physically connects to the pH circuit, built on a low-cost, easy-to-assemble breadboard integrating six pH Atlas-Scientific circuits to allow for the connection of up to six individual pH probes for the execution of up to six Chi.Bio reactors in parallel (Figure 1C).

For the circuitry integration, the original Chi.Bio reactor system includes an expansion header on the Main Reactor unit as shown circled in Figure 1B. The expansion header provides positive voltage, ground, and data connections to interface with the Chi.Bio controller (Figure 1D). Since data transmission occurs over the SDA^4^ and SCL^5^ pins using the I2C communication protocol, any I2C device can be implemented over this interface. Connection to this header was made using a 5-position mini-CT connector (Digikey). The EZO pH Circuit and Electrically Isolated EZO Carrier Board (Atlas Scientific) were selected as the pH transmitter using an off-the-shelf pH probe sourced from (ThermoScientific). The Carrier Board was connected to the Chi.Bio main reactor according to the wiring diagram in Figure 1E, where RX and TX are equivalently SCL and SDA, respectively, per the manufacturer documentation. Connections were made using a breadboard (Sparkfun) for rapid prototyping. The 12 V pin could be used to power the pH circuit if a voltage regulator was included. Instead, for simplicity, a voltage regulator was not implemented, and a separate power supply (Arduino Nano) was used to provide 3.3 V to the EZO Carrier Board to ensure the combined setup did not draw excessive current from the Chi.Bio 3.3 V power rail. To turn off the EZO Carrier Board, the OFF pin can optionally be connected to GND. In this implementation, the OFF pin was left unconnected. Before installing the EZO pH Circuit in the system, the EZO was switched from serial communication to the I2C protocol by following the protocol selection guidance in the Atlas Scientific EZO documentation. The source files for all hardware pieces are available in the Beckham-lab GitHub (*vide infra*).

### Software Integration of pH Control Functionality to Chi.Bio Reactors

To achieve stable pH control in the Chi.Bio system, the pH of the reactor is compared to a target pH value, up to a set tolerance, and this is repeated at regular time intervals that we refer to as the cycle time. If the pH is outside of the set range, a fixed volume of acid or base is added to the system every 90 s to bring the pH back within the set limits. The cycle time is a user-defined quantity to allow for experiments to incorporate a variety of neutralizing agents and reaction conditions. In the following paragraphs, we discuss the details of how this feedback mechanism is incorporated into the existing Chi.Bio software.

**Figure 2:**
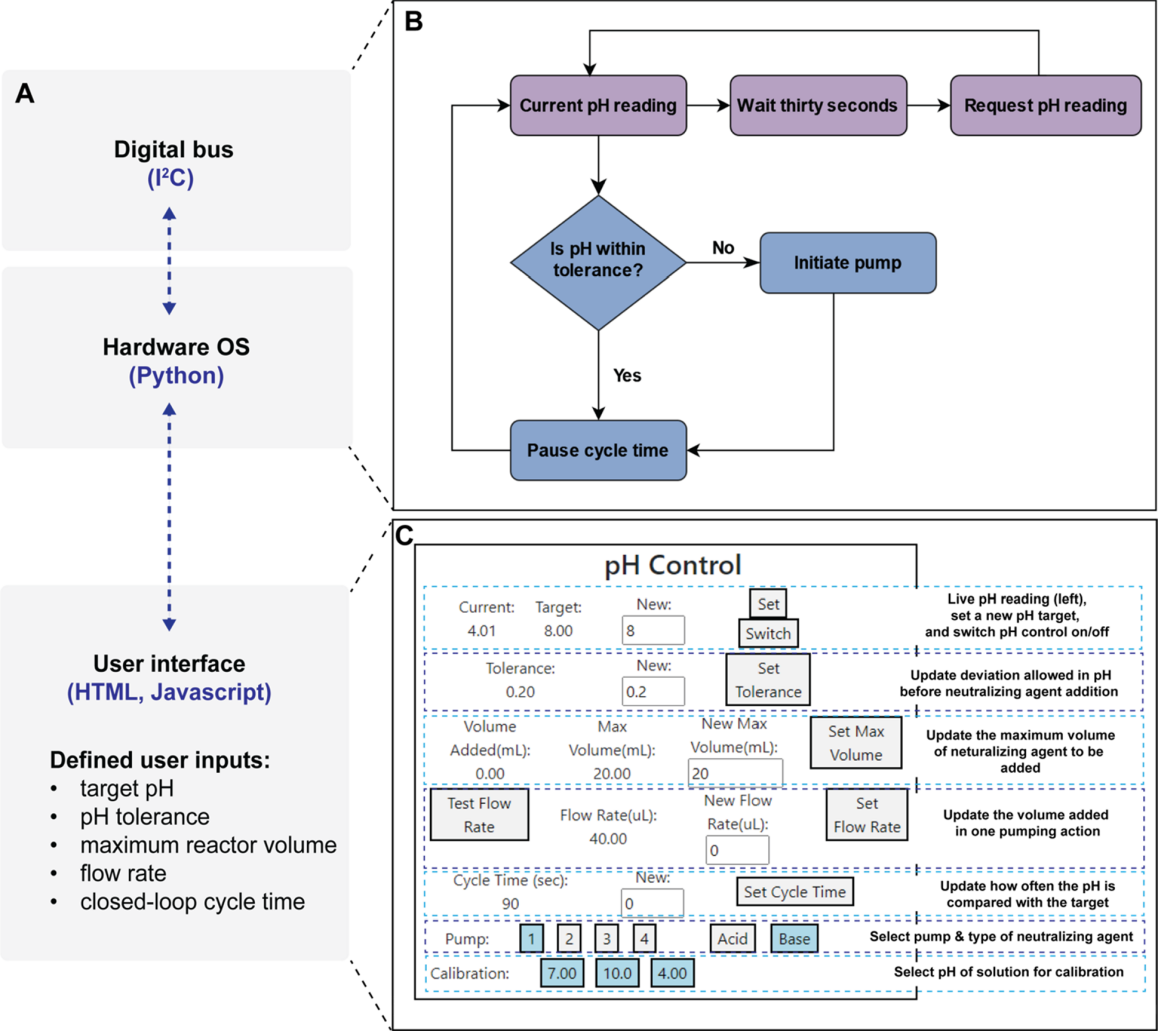
The software integration for pH control into Chi.Bio. (A) Overview of the Chi.Bio code that links the digital bus to the hardware OS and the live user interface. (B) Decision tree illustrating the function integrated into the digital bus and hardware OS to measure pH (upper horizontal flow cycle) and the function for the control of pH according to a predefined tolerance of a pH set point (lower cycle). (C) pH module embedded into the existing user Chi.Bio interface by modification to the HTML code.

The existing Chi.Bio system provides a user interface built in HTML/JavaScript that is accessible from a web browser and enables the user to have real-time control and monitor the experiment from a connected PC or network (Figure 2A). Behind the user interface, the Atlas Scientific EZO pH circuit was integrated into the existing I^2^C digital bus of Chi.Bio (Figure 2B). The Chi.Bio user interface was edited in HTML/JavaScript to incorporate a pH-control module (Figure 2C). The real-time measured pH is output to the UI Web server every 30 s to display the live pH value to the user (Figure 2C). Several tunable parameters were incorporated into the pH-control module in the form of clickable buttons, namely three buttons to calibrate the pH probe to pH 4, 7, and 10, one to select the desired pump line connected to the neutralizing agent (options 1–4), one to specify acid or base as the input, and one to activate the specified pump for the purpose of testing the flow rate. Additionally, three custom-input boxes were added to allow for the specification of the target pH, tolerance, and closed-loop control cycle time (Figure 2C). Finally, to prevent reactor overflow, the user can specify the maximum volume of neutralizing agent to be added to the reactor in mL, as a built-in safety feature.

The Python code linking the I^2^C digital bus to the real-time user interface was altered to introduce a closed-loop control on the pH inside the reactor as measured by the pH probe (Figure 2B). Specifically, a function was written to measure the pH every 30 s (Figure 2B). A separate function was added to compare the current pH reading to a user-defined pH range (determined by pH ± tolerance) after the cycle time has elapsed. If the current pH reading lies outside the tolerated range, the pump is engaged to add a fixed volume of acid or base necessary to bring the pH within range (Figure 2B). The fixed volume is the volume added by a single on/off activation of the selected pump and input line. To determine the fixed volume, a “test flow rate” button was included (Figure 2C). The volume added by the execution of this single on/off pump command corresponds to the fixed volume that will be added after each cycle time has elapsed. We manually determined the fixed volume to range between 30 and 45 ± 10 μL across individual Chi.Bio pumps for the setup and application we detail below. The fixed volume achieved by a single on/off pump activation can additionally vary according to the specific neutralizing agent. For any given pump, input line, and neutralizing agent, it is recommended to repeat the measurement for an accurate distribution of fixed volume fluctuation, and to specify the fixed volume using the “set flow rate” input box prior to each experiment. As our controller adds a fixed amount of liquid, in cases of large deviation from the pH set-point it might take multiple cycles of acid or base addition to return to the region of the set-point. This control approach is equivalent to an on–off controller (also known as *bang–bang*) with dead-zone, an effective and robust architecture often used in process control.^20^ Furthermore, this approach can be simplified and adapted for one-sided control (i.e., adding only acid or base during an experiment) when applied to regulate processes that drift toward high or low pH over time, as in the application detailed below.

The live pH readings are taken using the existing embed functionality in the Atlas Scientific pH circuit to correct for the temperature effect on pH. To ensure the pump adds the smallest possible volume of neutralizing agent, the pulse-width modulation was changed from 100% to 90%. Additionally, a custom function was written to block all other traffic on the I^2^C bus when the pumps are activated to ensure there is no delay in the off command being sent to the activated pump. The number of times the pump is activated in response to a change in pH is tracked and used to calculate the total volume of neutralizing agent added over the course of the experiment. For this calculation, a “flow rate” needs to be measured and inputted by the user. Each experiment generates a comprehensive log file of the measured pH values and corresponding cumulative volume of neutralizing agent added.

### Applying the pH Control Functionality to Enzymatic PET Deconstruction

To demonstrate the utility of integrating pH control into the Chi.Bio platform, enzymatic hydrolysis reactions of poly(ethylene terephthalate) (PET) were carried out using the modified bioreactors relative to control reactions in 1-L scale Applikon bioreactors with a 250 mL working volume for the reaction, with the aim of demonstrating reactor-to-reactor reproducibility. Such control for the *in vitro* pH regulation of enzyme-mediated PET depolymerization reactions could enable rapid benchmarking and comparison of PET hydrolases for deconstruction across a wide range of industrially relevant reaction conditions and facilitate enzyme engineering.^18,21,22^ Namely, a major limitation of microplate format directed evolution and enzyme engineering campaigns is accumulation of the terephthalic acid product, which limits the extents of PET conversion at industrially relevant solid loadings.^18^ For example, phenol red dye-based indicator assays have been successfully applied for pH-sensitive monitoring of the soluble terephthalate and acidic oligomer products released by PETase activity, yet these approaches are also hampered by a lack of pH control.^23^

**Figure 3:**
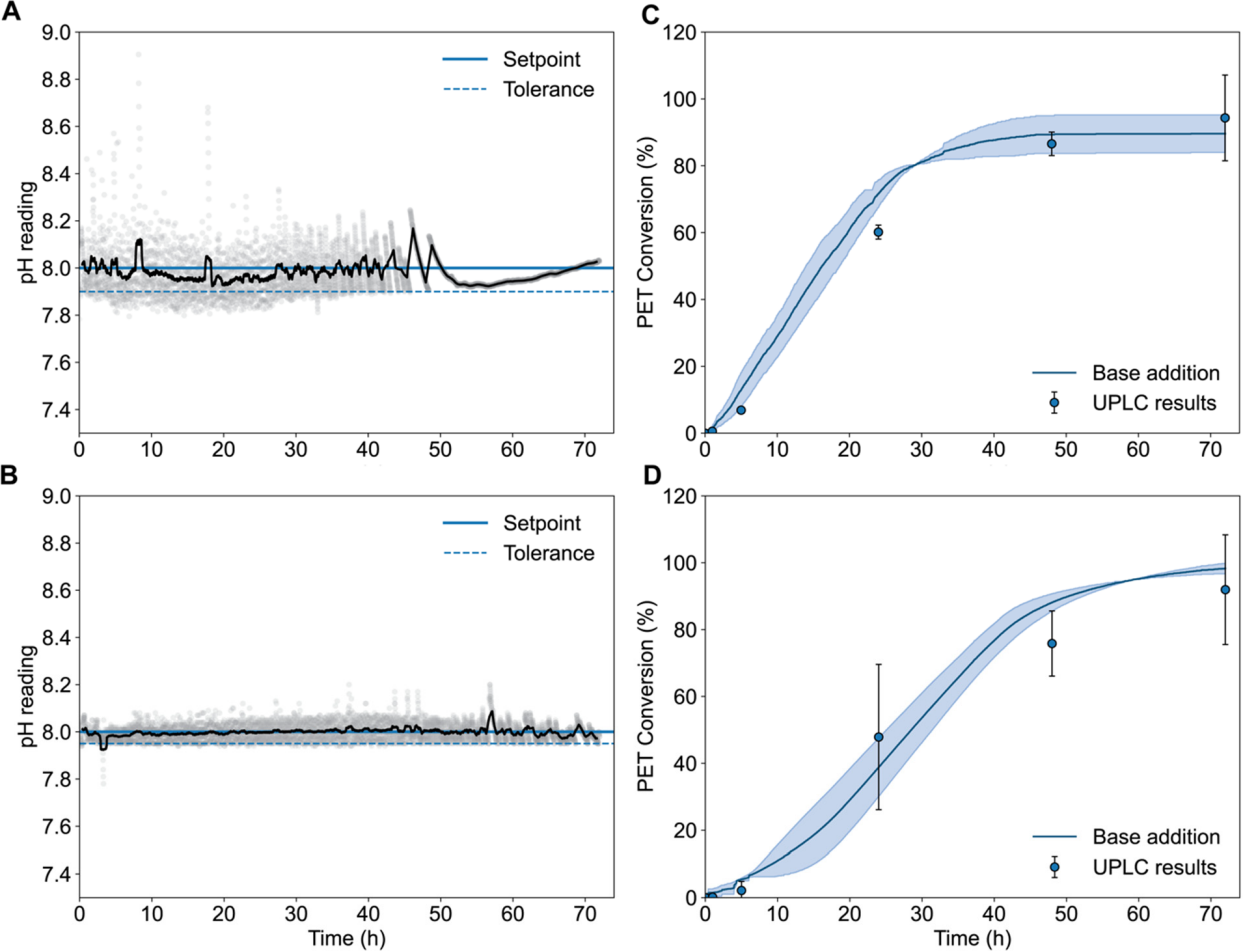
(A) Representative pH profiles with a rolling average pH calculated per 50 data points overlaid (black line) over all data points (light gray circles) in the Chi.Bio reactors, and (B) the Applikon bioreactors. The pH set point of 8 is shown as a solid blue line, and the respective tolerances (1 and 0.05 units below pH 8) as dashed-blue lines. (C) Extent of PET conversion as a function of time by PET hydrolase LCC^ICCG^ in the Chi.Bio reactors and (D) the Applikon bioreactors. The base addition extents of conversion (blue line) were calculated by sodium hydroxide addition, assuming that 2 mol of NaOH titrates 1 mol of TPA. The blue shaded zone represents the standard deviation of the calculated extent of PET conversion. Blue dots with error bars represent conversion based on UPLC quantification of TPA equivalents. The error bars denote the range of duplicate measurements.

Here, sodium hydroxide was used to neutralize the terephthalic acid product in real time of deconstruction of amorphous Goodfellow PET films by the ICCG variant of leaf-branch compost cutinase (LCC, UniProtKB G9BY57) (hereafter LCC^ICCG^),^24^ using the modified pH control functionality in the Chi.Bio system. Besides working volumes, the reaction conditions were identical between the Chi.Bio and Applikon reactor set-ups, where reactions were run in duplicate at 65 °C with a 10% mass loading of Goodfellow PET film (ES301445), and an enzyme loading of 3 mg enzyme/g PET, in 100 mM sodium phosphate buffer at pH 8. To establish that the Chi.Bio pH system could function for longer reaction times than 24 h,^18^ and specifically test the pH probes under extended heating at 65 °C, amorphous PET films were used in depolymerization reactions run for 72 h. The Chi.Bio reactors are 2.5 cm × 9.5 cm, and therefore, the PET film size was adjusted to 1 cm × 1 cm to fit in the vials, as compared to the 2.5 × 2.5 cm films used in the Applikons. The ratio of difference in reactive surface area of the two film sizes was small (1.25:1). The reactions achieved maintenance of pH control by applying a 0.1 pH unit tolerance range of the pH 8 target in the Chi.Bio reactors, and a 0.05 tolerance in the Applikon setup (Figure 3). We chose 0.1 for the Chi.Bio pH regulation as this is the maximum limit of precision of the integrated ThermoScientific Orion Economy Series pH Combination Electrode. Across the two Chi.Bio reactors, the pH was maintained within 0.1 units of the target pH 8 for 78.7% and 80.2% of the total 72-h reactions, respectively, compared to 95.8 and 98.6% of the total reaction time in the Applikon reactors (Figure 3, Data Sets S2–S3).

After 72 h, the contents of the reactors were filtered and dried to collect any residual PET solids. The final dried filtered solids were weighed to determine the final mass loss and used to calculate the extent of conversion of 92.6 ± 1.67% of PET in the Chi.Bio system, compared to 95.5 ± 2% in the Applikon reactors (Table 1). A conversion of 94.3 ± 12.8% was achieved as measured by ultrahigh-performance liquid chromatography (UPLC) time point sampling in Chi.Bio by 72 h, compared to a final 92 ± 16.4% in the Applikon bioreactors by 72 h, respectively (Figure 3). At the 24 h time point, the soluble monoacid product mono(2-hydroxyethyl) terephthalate (MHET) comprised 9.8% of the total aromatic product sum measured in the Chi.Bio reactors, which was all converted to TPA by 48 h (Data Set S4). Comparatively, MHET was not detected in the Applikon reactor samples under these enzyme and solid loadings (Data Set S5), although MHET accumulation profiles can vary significantly according to reaction conditions and PET substrate selection.^25^ The error in the UPLC results of the duplicate Applikon reactors was demonstrably larger compared to the Chi.Bio UPLC results (Figure 3D). This may be attributed to a more efficient mixing in the smaller volume of the Chi.Bio reactors and therefore a more homogeneous sampling of the heterogeneous mixture of the PET solids, terephthalic acid, and ethylene glycol products between duplicate reactors.

**Table 1:** Yield Quantification Measured by Mass Loss and UPLC for PET Degradation in Chi.Bio and Applikon Bioreactors

	Chi.Bio reactors	Applikon reactors
Initial PET mass (g)	1.2	25
Final PET mass (g)	0.089 ± 0.16	1.1 ± 0.50
Extent of conversion by mass (%)	92.6 ± 1.67	95.5 ± 2.02
Extent of conversion by UPLC (%)	94.3 ± 12.8	92.0 ± 16.4

While the final PET mass loss was similar in both systems (Table 1), a difference in the plateau of the rate of reaction was evident by comparison of the pH and base conversion profiles (Figure 3). These results suggest that pH tolerance values should be reported as part of standardization for comparison between studies of pH-controlled enzymatic PET deconstructions, as varying yield profiles may result from the application of different tolerances. Factors to explain the rate differences may include the differences in pH fluctuations, different surface area-to-volume ratios of the two reactor setups, or the different agitation schemes. In the Applikon reactors, constant agitation was applied at 400 rpm, compared to the existing stirring cycle time implemented in the Chi.Bio reactors which pauses every 60 s for data collection. Agitation rates of the enzyme and substrate, and corresponding shear stresses can also dictate enzyme stability over time in a reactor.^26^

## Discussion

In this work, we implemented pH control software and hardware modifications into the Chi.Bio platform and demonstrated the utility of this modification for small-scale PET enzymatic hydrolysis reactions. We present this as a cost-effective platform that could be used to accelerate the evaluation of PET hydrolases in industrially relevant conditions. There has been a rapid expansion in the number of PET hydrolases and improved variants reported in the literature in the last 5 years alone.^27−29^ However, there is a need in the field to establish benchmarking of activity of the potentially process relevant PET hydrolases at industrially relevant loadings.^18,30,31^ In our results, we compared the robustness of the pH control in Chi.Bio to that implemented in Applikon bioreactors and established maintained conversion rates at the smaller scale, highlighting reproducibility in scaling-down base-controlled PET hydrolase reactions. This cost-effective system adds to a growing number of automated microreactor systems.^32^ It also opens opportunities to dissect a wide variety of polymer substrates in deconstruction reactions without the requirement for gram to kilogram-scale substrate quantities and enables significant reductions in the purified enzyme required.

There are a variety of potential modifications that could further enhance the pH functionality we have integrated into the Chi.Bio system. For example, the pH probe that was integrated was the ThermoScientific Orion Economy Series pH Combination Electrode. Future modifications could incorporate a glass membrane pH probe, with an external glass electrode body, which may be better suited to repeated applications requiring high temperature or chemical resistance, and with greater precision than the 0.1 pH unit of precision of the existing probe. In the event of the integration of an alternative probe, the dimensions of the head plate could be correspondingly modified. The system utilizes the native Chi.Bio pump board, but could be swapped out for alternatives, such as a syringe pump, according to the sensitivity of regulation required. For microbial growth applications, the modified system is currently best suited to batch fermentations, or those requiring only a single-input line for neutralizing agent addition. Oxygen agitation for fermentation can be achieved with the existing Chi.Bio stirring function, and future modifications could also benefit from the integration of a dissolved oxygen stat module for aerobic cultivations. For the enzymatic depolymerization application shown here, the mass of the neutralizing agent and reactors before and after each enzymatic deconstruction was manually measured and tracked. The future integration of programmable microscales would allow for real-time monitoring of the neutralizing agent added and accurate quantification of reaction mass. Finally, the system could be applied in conjunction with robotic systems. Similar studies have integrated the original Chi.Bio platform to the low-cost Opentrons robotic liquid-handling systems for alternative applications.^33^

The 3.3 V output on the Arduino nano utilized in our pH control platform can supply up to 50 mA. As each Atlas scientific pH module has a maximum power consumption of 14.5 mA (with the LED off), and a sleep power consumption of 1 mA, this dictates that ∼3 pH Atlas Scientific modules can measure pH simultaneously. With a sleep current of 1 mA, in theory, ∼45 pH units could be powered from a single Arduino, with staggered read times in the software to save power, where no more than 3 would be used simultaneously. At a cost of $23 per Arduino nano at the time of writing, one Arduino nano per 3 pH probes is an appealing potential opportunity for scaling up the number of pH-controlled reactions that can be run in parallel in this system. Alternatively, a dedicated power supply could be used to replace the Arduino nano, provide increased current at 3.3 V, and enable further parallelization. However, there will also be practical and cost considerations associated with scale-up. We estimate that one experimentalist may be able to operate and manage ∼20 reactors in parallel, each potentially testing different conditions. In total, 8 Chi.Bio reactors can be connected per Beaglebone control computer. Therefore, for an example case of 20 pH-controlled Chi.Bio reactors, 3 Beaglebones and 3 Arduinos could be used. Overall, at a total cost of ∼$5k for all components per 4 reactors, the Chi.Bio pH platform is ∼40-fold cheaper than the ∼$180–240k typical price of 4 pH-controlled, liter-scale reactors.

## Conclusions

Overall, we have demonstrated the successful enzymatic deconstruction of PET with pH control in scaled-down PET hydrolase reactions. The reactions demonstrated are a single application of the pH module we have integrated into the Chi.Bio platform. The functionality implemented should be broadly useful for a wide variety of biotechnological, biochemical, and synthetic biology applications including multienzymatic cascade reactions and pH-stat controlled growth of engineered strains of micro-organisms. The full range of biologically accessible pH values with corresponding control can be utilized for these applications using the Chi.Bio platform.

## Experimental Procedures

### Protein Expression and Purification

LCC^ICCG^ (LCC, Uniprot Accession ID: G9BY57) DNA was synthesized (Twist Bioscience) and cloned into a pET-21b(+) expression vector (EMD Biosciences) as previously described.^25^ The plasmid was transformed into OverExpress *Escherichia coli* C41 (DE3) (Lucigen) cells, plated on lysogeny broth (LB)-agar plates containing 100 μg/mL ampicillin (Amp), and incubated at 37 °C overnight. A single colony from transformation was inoculated into a starter culture of LB liquid media containing 100 μg/mL Amp and cultures were grown at 37 °C, 250 rpm overnight. The starter culture was inoculated at a 100-fold dilution in 2× YT media containing 100 μg/mL Amp and grown at 37 °C to OD_600_ = 0.6–0.8. Protein expression was induced by the addition of isopropyl β-D-1-thiogalactopyranoside (IPTG) at 1 mM. Cells were maintained at 18 °C, 225 rpm for 20 h following induction, harvested by centrifugation, and stored at −80 °C until purification. Harvested cells were resuspended in lysis buffer (20 mM Tris pH 8, 10 mM imidazole, 300 mM NaCl, 1 mg/mL lysozyme, 50 μg/mL DNAase) and subjected to sonication (QSonica Q700). The resulting lysate was clarified by centrifugation at 40,000 × *g* for 40 min at 4 °C. The clarified lysate was applied to a 25 mL HisTrap HP (Cytiva) column linked to a ÄKTA Pure chromatography system (Cytiva) and eluted with elution buffer (20 mM Tris pH 8, 500 mM imidazole, 300 mM NaCl) over a 2 CV gradient. The fractions containing the protein were dialyzed overnight into a 20 mM Tris, pH 8, 300 mM NaCl buffer, and the protein purity confirmed by SDS-PAGE. The concentration was determined by 280 nm absorbance readings and calculated using an extinction coefficient of 37150 M^–1^ cm^–1^.

### Chi.Bio Bioreactor PET Deconstruction

Bioreactor hydrolysis reactions were performed in the Chi.Bio reactors with a reaction volume of 12 mL, with a 0.25 mm thick Goodfellow PET film (ES301445) cut into 1 cm × 1 cm squares as the substrate. For the reactions, 1.2 g of PET substrate (1.38 mL volume based on density of PET) was added to 100 mM phosphate pH 8 assay buffer at a final volume of 12 mL and equilibrated to 65 °C with a stirring rate set to 0.2. The reactions were initiated by the addition of 0.77 mL of 4.7 mg/mL LCC-ICCG for a final enzyme loading of 3 mg/g PET. Reactions proceeded for 72 h and were maintained at pH 8 with 1 M NaOH addition using the integrated pH functionality. Sample volumes of 0.1 mL were removed at designated time points, quenched with an equal volume of methanol, stored, and filtered. At the end of the reaction time course, the remaining substrate was collected by filtration through Whatman grade 2 filter paper (Cytiva) and a Büchner funnel. The filters were preweighed, and the filters with PET were dried for 3 days at 40 °C under vacuum before the final mass of residual PET was calculated.

### Applikon Bioreactor PET Deconstruction

Bioreactor hydrolysis reactions were performed at 0.25 L scale in duplicate in 1 L glass bioreactors (Applikon Biotechnology), which included two Rushton impellers in the stirrer shaft below the 200 mL line. The substrate used was Goodfellow PET film (ES301445) cut into 2.5 cm × 2.5 cm squares. For the reactions, 25 g of PET substrate (18 mL volume based on density of PET) was added to 100 mM phosphate pH 8 assay buffer at a final volume of 0.25 L and equilibrated to 65 °C with stirring at 400 rpm. The reactions were initiated by the addition of 16 mL of 4.7 mg/mL LCC-ICCG for a final enzyme loading of 3 mg/g PET. Reactions proceeded for 72 h and were maintained at pH 8 with 4 M NaOH addition using a peristaltic pump controlled by an in-control module (Applikon Biotechnology). Sample volumes of 0.5 mL were removed at designated time points, quenched, stored, and filtered. At the end of the reaction time course, the remaining substrate was collected by filtration through Whatman grade 2 filter paper (Cytiva) and a Büchner funnel. The filters were preweighed, and the filters with PET were dried for 3 days at 40 °C under vacuum before the final mass of residual PET was calculated.

### UPLC Quantification

Analysis of aromatic products MHET, BHET, and TPA was performed by ultrahigh performance liquid chromatography (UHPLC) as previously described.^22^ Briefly, samples were injected onto a Zorbax Eclipse Plus C18 Rapid Resolution HD column, and separation was achieved using a mobile phase gradient of 20 mM phosphoric acid and methanol. Diode array detection (DAD) was utilized for quantitation for the analytes of interest using a wavelength of 240 nm.

## Data Availability

Code for the pH control for Chi.Bio and the source files for all hardware pieces are available in the Beckham-lab GitHub at https://github.com/beckham-lab/Chi.Bio.pH.

## Supporting Information Available

List of all pH module hardware components (PDF)
Data sets displaying base consumption and conversion profiles of bioreactor reactions, UPLC data sets for bioreactor reactions (XLSX)

## Accession Codes

LCC^ICCG^ (LCC, Uniprot Accession ID: G9BY57).
