# Supplementary material for: Integration of pH Control into Chi.Bio Reactors and Demonstration with Small-Scale Enzymatic Poly(ethylene terephthalate) Hydrolysis
Source: Biochemistry. 2024 Jun 22;63(13):1599–607. doi: 10.1021/acs.biochem.4c00149 (PMC11223484; doi:10.1021/acs.biochem.4c00149)
Supplement: Supplementary file 1 — bi4c00149_si_001.pdf [file bi4c00149_si_001.pdf]

Supporting Information for:

Integration of pH control into Chi.Bio reactors and demonstration with small-scale enzymatic poly(ethylene terephthalate) hydrolysis

Mackenzie C.R. Denton,[1,2,‡] Natasha P. Murphy,[1,2,‡] Brenna Norton-Baker,[1,2] Mauro Lua,[3] Harrison Steel,[4] Gregg T. Beckham[1,2,*]

1. Renewable Resources and Enabling Sciences Center, National Renewable Energy Laboratory, Golden, 80401, CO, USA
2. BOTTLE Consortium, Golden, CO, 80401, USA
3. Catalytic Carbon Transformation and Scale-up Center, National Renewable Energy Laboratory, Golden, 80401, CO, USA
4. Department of Engineering Science, University of Oxford, Oxford, OX1 3PJ, UK

‡ Denotes equal contribution

* Correspondence: gregg.beckham@nrel.gov

S1: Hardware components for the pH system integrated into the Chi.Bio platform.

S2: Base profile and base conversion dataset for enzymatic PET deconstructions in Chi.Bio bioreactors.

S3: Base profile and base conversion dataset for enzymatic PET deconstructions in Applikon bioreactors.

S4: UPLC dataset for enzymatic PET deconstructions in Chi.Bio bioreactors.

S5: UPLC dataset for enzymatic PET deconstructions in Applikon bioreactors.

Table S1: Hardware components for the pH system integrated into the Chi.Bio platform.

**Hardware Component**	**Source**
Chi.Bio reactors, control computer and pump set.	https://www.labmaker.org/products/chi-bio
Orion™ Economy Series pH Combination Electrode (Part No. #911600)	https://www.thermofisher.com/order/catalog/product/911600
Air-Tite™ Premium Hypodermic Needles for Lab/Vet Use needle	https://www.fishersci.com/shop/products/premium-hypodermic-needles-lab-vet-use/p-2537820
AlteSil High Strength Tubing, Altec (Part No. #01-93-1416)	https://altecweb.com/home.asp?cat=category1201
EZO pH Circuit (Part No. #EZO-pH)	https://atlas-scientific.com/embedded-solutions/ezo-ph-circuit/
Electrically Isolated EZO Carrier Board (Part No. #ISCCB-2)	https://atlas-scientific.com/carrier-boards/electrically-isolated-ezo-carrier-board-gen-2/<br>For wiring diagram, refer to documentation datasheet: https://atlas-scientific.com/embedded-solutions/ezo-ph-circuit/
Breadboard (Part No. #12615)	https://www.sparkfun.com/products/12615
Arduino nano (Part No. #A000005)	https://store.arduino.cc/products/arduino-nano
5-position mini-CT connector (Part No. #A100195-ND)	https://www.digikey.com/en/products/detail/te-connectivity-amp-connectors/2058943-4/2136139
3D pH-modified head plate.	.STL CAD design file at: https://github.com/beckham-lab/Chi.Bio.pH
